# Simultaneous Analysis of Losartan Potassium, Amlodipine Besylate, and Hydrochlorothiazide in Bulk and in Tablets by High-Performance Thin Layer Chromatography with UV-Absorption Densitometry

**DOI:** 10.1155/2012/108281

**Published:** 2012-04-08

**Authors:** Karunanidhi Santhana Lakshmi, Sivasubramanian Lakshmi

**Affiliations:** Department of Pharmaceutical Analysis, SRM College of Pharmacy, SRM University, Tamilnadu, Kattankulathur 603 203, India

## Abstract

A Simple high-performance thin layer chromatography (HPTLC) method for separation and quantitative analysis of losartan potassium, amlodipine, and hydrochlorothiazide in bulk and in pharmaceutical formulations has been established and validated. After extraction with methanol, sample and standard solutions were applied to silica gel plates and developed with chloroform : methanol : acetone : formic acid 7.5 : 1.3 : 0.5 : 0.03 (*v*/*v*/*v*/*v*) as mobile phase. Zones were scanned densitometrically at 254 nm. The *R*_*f*_ values of amlodipine besylate, hydrochlorothiazide, and losartan potassium were 0.35, 0.57, and 0.74, respectively. Calibration plots were linear in the ranges 500–3000 ng per spot for losartan potassium, amlodipine and hydrochlorothiazide, the correlation coefficients, *r*, were 0.998, 0.998, and 0.999, respectively. The suitability of this method for quantitative determination of these compounds was by validation in accordance with the requirements of pharmaceutical regulatory standards. The method can be used for routine analysis of these drugs in bulk and in formulation.

## 1. Introduction

Losartan (LOS), 2-n-butyl-4-chloro-5-hydroxy methyl-1-[2′-(1H-tetrazol-5-yl) (biphenyl-4-yl) methyl] imidazole, potassium is a strong nonpeptide antihypertensive agent which exerts its action by specific blocking of angiotensin II receptors [1]. It has a gradual long-lasting effect as an antihypertensive. Amlodipine (AML), 3-ethyl 5-methyl (RS) 2-(2-aminoethoxymethyl)-4-(2-Chlorophenyl)-6-methyl-dihydropyridine-3,5-dicarboxylate is a calcium channel blocker which inhibits the influx of extracellular calcium across the myocardial and vascular muscle cell membranes [2]. Hydrochlorothiazide (HCZ), 6-chloro-3,4-dihydro-2H-1,2,4-benzothiadiazine-7-sulphonamide 1,1-dioxide, which is widely used in antihypertensive pharmaceutical preparations reduces active sodium reabsorption and peripheral vascular resistance [1–3]. 

A literature survey reveals that a variety of spectrophotometric and chromatographic methods, including UV derivative, the simultaneous equation method, colorimetric determination, HPLC, ratio derivative and compensation technique, and a stability indicating HPLC method, have been reported for the determination of LOS in pharmaceutical dosage forms in combination with other drugs [4–14]. Spectrophotometric and chromatographic methods have been reported for determination of AML, in combination with other drugs, in bulk and pharmaceutical dosage forms [15–24]. A variety of methods have been used for the determination of hydrochlorothiazide [25–34]. No method has been reported for the simultaneous estimation of LOS, AML, and HCZ in the combined dosage form.

In recent years TLC has been improved to incorporate HPTLC-grade stationary phases, automated sample-application devices, a controlled development environment, automated development, forced-flow techniques, computer-controlled densitometry, quantitation, and fully validated procedures. These features result in methods which are not only convenient, rapid, robust, and cost effective but also reproducible, accurate, and reliable. The objective of this investigation was, therefore, to establish an HPTLC method for simultaneous estimation of LOS, AML, and HCZ in bulk and in tablets.

## 2. Experimental

### 2.1. Materials and Reagents

 Analytically pure samples of losartan potassium, hydrochlorothiazide, and amlodipine besylate were procured from Madras Pharmaceuticals, Chennai, as gift samples and used as working standards, methanol of HPLC grade from Merck (Mumbai, India), and chloroform, acetone, and formic acid of analytical reagent grade from S.D. Fine Chemicals were used, without purification to prepare the mobile phase.

 A solution containing 1 mg/mL losartan potassium, amlodipine besylate, and hydrochlorothiazide was prepared by dissolving 10 mg of each standard in 10 mL methanol and was used as working standard solution.

### 2.2. Sample Preparation

Twenty TRILOPACE* tablets by Akums Drugs & Pharmaceuticals Ltd containing 50 mg losartan potassium, 5 mg amlodipine besylate, and 12.5 mg hydrochlorothiazide were weighed and powdered. An amount of powder equivalent to 50 mg of LOS, 5 mg of AML, and 12.5 mg of HCZ was transferred to a 50 mL volumetric flask. After addition of 30 mL of methanol and sonication (30 min), the solution was diluted to volume with the same solvent and filtered through a 0.45 *μ* filter (Millipore, Milford, MA, USA). To this solution known amount of amlodipine standard (20 mg) was added (standard addition method) as its content is very low in the formulation. This solution (1.0, 2.0, and 3.0 *μ*L containing 1000, 2000, and 3000 ng/spot of LOS, 500, 1000, and 1500 ng/spot of AML, and 250, 500, and 750 ng/spot of HCZ) was used for assay of losartan potassium, amlodipine, and hydrochlorothiazide in the tablets.

### 2.3. Chromatography

 Chromatography was performed on 10 cm × 10 cm aluminium HPTLC plates coated with 0.2 mm layers of silica gel 60 F_254_ (Merck). Samples were applied as 6 mm bands by means of a CAMAG (Muttenz Switzerland) Linomat V automatic sample applicator equipped with a 100 *μ*L syringe (Hamilton, Reno, Nevada, USA). The distance between the bands of 14.0 mm and the spraying rate of 50 nl per second were maintained. Ascending development of the plate up to a distance of 85 mm was performed at 25 ± 2°C, with chloroform-methanol-acetone-formic acid 7.5 : 1.3 : 0.5 : 0.03 (*v*/*v*/*v*/*v*) as mobile phase. The development was carried out in a CAMAG twin-trough chamber previously saturated with mobile phase vapour for 20 min. The average development time was 30 min. Densitometric scanning at 254 nm was performed with a CAMAG TLC scanner 3 equipped with CAMAG Wincats software version 1.4.4 using deuterium light source. During scanning process, the slit dimensions were fixed at 4.00 mm × 0.30 mm.

## 3. Results and Discussion

### 3.1. Validation of the Method

 The method was validated in accordance with ICH guidelines [35].

#### 3.1.1. Linearity

 Different aliquots of standard solution equivalent to 0.5–3 *μ*g of LOS, AML, and HCZ per band were applied on the precoated TLC plates. The plates were then developed, dried, and scanned as described above. Calibration plots were constructed by plotting peak areas against the corresponding concentration of drugs (ng per spot). For all three drugs, the detector response was found to be a linear function of amount in the range 500–3000 ng per spot. The correlation coefficients of all the three drugs were found to be 0.9988 for LOS, 0.9985 for AML, and 0.9990 for HCZ, respectively. The average linear regression equations were *Y* = 4571.56*X* + 416.48  for LOS, *Y* = 3229.40*X* + 300.24 for AML, and *Y* = 5917.03*X* + 422.70  for HCZ.

#### 3.1.2. Sensitivity

 The sensitivity of measurement of LOS, AML, and HCZ was estimated in terms of the limit of quantitation (LOQ). The smallest amount of each drug was also detected under the chromatographic conditions in terms of the limit of detection (LOD). LOQ and LOD were calculated by use of the equations LOD = 3 × *N*/*B* and LOQ = 10 × *N*/*B*, where *N* is the standard deviation of the peak areas of the drugs, taken as a measure of the noise and *B* is the slope of the corresponding calibration plot. LOQ and LOD for losartan potassium were found to be 0.382 and 0.121 *μ*g/spot, respectively. For amlodipine they were 0.584 and 0.188 *μ*g/spot, respectively, and for hydrochlorothiazide they were 0.497 and 0.162 *μ*g/spot, respectively.

### 3.2. Evaluation of Precision for Assay of the Pharmaceutical Preparation

 The amount of losartan potassium, amlodipine, and hydrochlorothiazide in the pharmaceutical preparation were determined by replicate analysis (*n* = 3). The results are reported in Table 1.

Precision was determined by analysis of standard solutions containing concentrations of LOS, AML, and HCZ covering the entire calibration range. The precision of the method as intraday variation (CV, %) was determined by analysis of these solutions three times on the same day. Interday precision (CV, %) was assessed by analysis of these solutions on three different days over a period of one week. The results of the precision studies are shown in Table 2.

#### 3.2.1. Accuracy

 An accuracy of the method was determined by analysis of standard additions at three different levels, that is, multiple-level recovery studies. The preanalyzed sample solution (2, 0.5, and 0.2 *μ*g/mL of LOS, HCZ, and AML) was spiked with amounts equivalent to 80, 100, and 120% of standard drugs. These solutions were reanalysed, and the recoveries were found to be within the acceptable limits (Table 3).

#### 3.2.2. Specificity

**Figure 1:**
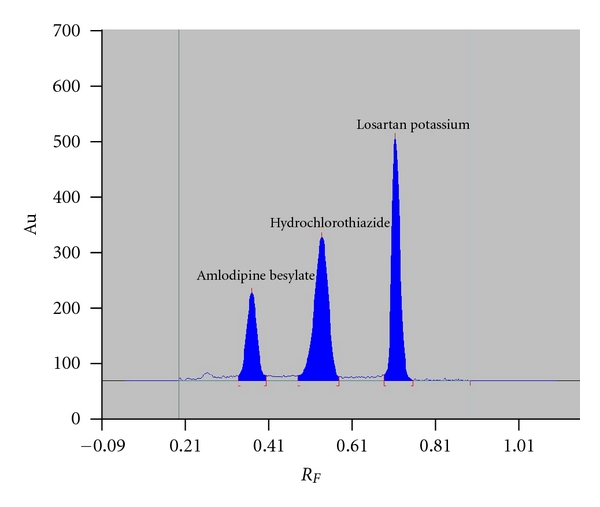
Typical densitogram obtained from losartan potassium (*R*_*F*_ = 0.74), amlodipine besylate (*R*_*F*_ = 0.35), and hydrochlorothiazide (*R*_*F*_ = 0.57). Detection was at 254 nm and the mobile phase was chloroform : methanol : acetone : formic acid 7.5 : 1.3 : 0.5 : 0.03 (*v*/*v*/*v*/*v*).

**Figure 2:**
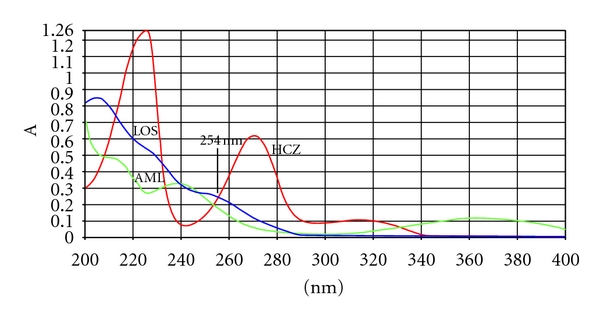
Typical absorption spectra of losartan potassium, amlodipine besylate, and hydrochlorothiazide.

 The mobile phase used was found to be effective in resolving the drugs (Figure 1). The *R*_*F*_ values of losartan potassium, amlodipine, and hydrochlorothiazide were 0.74, 0.35, and 0.57, respectively. Typical overlaid absorption spectra of LOS, AML, and HCZ is shown in Figure 2. Peak purity of the drugs was tested by acquiring spectra at the peak start (*S*), peak apex (*A*), and peak end (*E*) positions. Results from correlation of the spectra were for losartan potassium *r*(*S*, *M*) = 0.9996 and *r*(*M*, *E*) = 0.9994, for amlodipine *r*(*S*, *M*) = 0.9994 and *r*(*M*, *E*) = 0.9996, and for hydrochlorothiazide *r*(*S*, *M*) = 0.9998 and *r*(*M*, *E*) = 0.9997. The results of peak purity ensure the specificity and can conclude that no impurities or degradation products were coeluted.

#### 3.2.3. Repeatability

 The repeatability of sample preparation was assessed by application of 2 *μ*L standard drug solution six times on a HPTLC plate. After development of plate, peak height and peak area were recorded for the zones. The CV (%) of peak height and area were calculated and found to be 0.45 and 0.56, respectively, for LOS, 0.34 and 0.43 for AML, and 0.67 and 0.32 for HCZ. 

## 4. Conclusion

 The proposed HPTLC method for simultaneous analysis of losartan potassium, amlodipine, and hydrochlorothiazide in pharmaceutical dosage forms has been established for the first time. Use of HPTLC enables analysis of several samples at the same time. The method is very simple, rapid, and provides accurate and precise results.

## Figures and Tables

**Table 1:** Results from assay of losartan potassium, amlodipine besylate, and hydrochlorothiazide in Trilopace*H tablets.

Component	Label claim (mg)	Amount found (mg ± SD, *n* = 3)	Percentage of label claim (±SD)
Losartan potassium	50	49.51 ± 0.330	99.03 ± 0.665
Amlodipine besylate	5	4.86 ± 0.057	98.47 ± 1.114
Hydrochlorothiazide	12.5	12.4 ± 0.036	99.22 ± 0.284

**Table 2:** Results from evaluation of precision.

Drug	Concentration (ng per spot)	Intraday precision (CV, %, *n* = 3)	Interday precision (CV, %, *n* = 3)
Losartan potassium	1000	0.345	0.386
	2000	0.523	0.563
	3000	0.213	0.254

Amlodipine besylate	500	0.324	0.382
	1000	0.651	0.685
	1500	0.772	0.791

Hydrochlorothiazide	500	0.821	0.882
	1500	0.631	0.653
	2500	0.812	0.876

**Table 3:** Results from recovery studies.

Brand name	Drug	Recovery level (%)	Initial amount (ng)	Amount added (ng)	Recovery (%)	CV (%)
Trilopace*H	Losartan potassium (50 mg)	80	2000	1600	100.56	0.213
		100	2000	2000	99.43	0.321
		120	2000	2400	99.67	0.422
	Amlodipine besylate (5 mg)	80	200	160	100.58	0.616
		100	200	200	101.12	0.222
		120	200	240	99.85	0.414
	Hydrochlorothiazide (12.5 mg)	80	500	400	99.43	0.552
		100	500	500	99.21	0.608
		120	500	600	100.63	0.621
